# Synergistic Effects of Radical Distributions of Soluble and Insoluble Polymers within Electrospun Nanofibers for an Extending Release of Ferulic Acid

**DOI:** 10.3390/polym16182614

**Published:** 2024-09-15

**Authors:** Ran Dong, Wenjian Gong, Qiuyun Guo, Hui Liu, Deng-Guang Yu

**Affiliations:** School of Materials and Chemistry, University of Shanghai for Science and Technology, Shanghai 200093, China; 232223031@st.usst.edu.cn (R.D.); 223353279@st.usst.edu.cn (W.G.); 233393129@st.usst.edu.cn (Q.G.); huiliu@usst.edu.cn (H.L.)

**Keywords:** radical distributions, soluble polymers, insoluble polymers, sustained release, coaxial electrospinning, core–shell nanofibers

## Abstract

Polymeric composites for manipulating the sustained release of an encapsulated active ingredient are highly sought after for many practical applications; particularly, water-insoluble polymers and core–shell structures are frequently explored to manipulate the release behaviors of drug molecules over an extended time period. In this study, electrospun core–shell nanostructures were utilized to develop a brand-new strategy to tailor the spatial distributions of both an insoluble polymer (ethylcellulose, EC) and soluble polymer (polyvinylpyrrolidone, PVP) within the nanofibers, thereby manipulating the extended-release behaviors of the loaded active ingredient, ferulic acid (FA). Scanning electron microscopy and transmission electron microscopy assessments revealed that all the prepared nanofibers had a linear morphology without beads or spindles, and those from the coaxial processes had an obvious core–shell structure. X-ray diffraction and attenuated total reflectance Fourier transform infrared spectroscopic tests confirmed that FA had fine compatibility with EC and PVP, and presented in all the nanofibers in an amorphous state. In vitro dissolution tests indicated that the radical distributions of EC (decreasing from shell to core) and PVP (increasing from shell to core) were able to play their important role in manipulating the release behaviors of FA elaborately. On one hand, the core–shell nanofibers F3 had the advantages of homogeneous composite nanofibers F1 with a higher content of EC prepared from the shell solutions to inhibit the initial burst release and provide a longer time period of sustained release. On the other hand, F3 had the advantages of nanofibers F2 with a higher content of PVP prepared from the core solutions to inhibit the negative tailing-off release. The key element was the water permeation rates, controlled by the ratios of soluble and insoluble polymers. The new strategy based on core–shell structure paves a way for developing a wide variety of polymeric composites with heterogeneous distributions for realizing the desired functional performances.

## 1. Introduction

Polymers and lipids are the two most popular excipients for encapsulating active pharmaceutical ingredients (APIs) [1,2,3]. Particularly, polymers with different properties have been broadly explored for providing a wide variety of drug controlled-release profiles [4,5,6,7]. In general, soluble polymers are frequently employed to promote the dissolution of poorly water-soluble drugs such as ketoprofen, paclitaxel, and asenapine maleate [8,9,10]. In contrast, insoluble polymers and biodegradable polymers are able to provide extended- or sustained-release profiles [11,12,13,14,15,16]. Other release profiles, such as delayed release, biphasic release, and multiple-stage release, can be provided by combinations of soluble/insoluble polymers, and even inorganic carriers [17,18,19,20,21]. One example is the biphasic release, which can be achieved simply by a blending of soluble and insoluble polymers [22]. Later, with the developments of new pharmaceutical techniques, the soluble and insoluble polymers can be integrated into one product, forming a series of complex structures to achieve biphasic release, such as core–shell nanofibers/microparticles [23], Janus fibers/particles [24], fiber–particle hybrids [25,26], and casting films containing particles [27].

Among all types of drug controlled-release profiles, sustained release is one of the most fundamental and popular, due to its ability to prevent potential toxicity, enhance the therapeutic effects, and improve patient compliance [28,29,30,31,32]. Initially, drug molecules are simply loaded into the insoluble polymers to extend drug release over a relatively longer period [33,34,35]. Soluble polymers can also be explored to provide sustained-release profiles, often cross-linking after the drug molecules are successfully encapsulated within the polymeric matrices [36]. Presently, with the advancements in nanoscience and nanoengineering, more complex nanostructures and strategies have been developed to manipulate the sustained-release profiles of loaded drug molecules [2,37,38]. These structures can be designed by manipulating a series of elements, such as components, compositions, and spatial distribution. Among all complex nanostructures, the core–shell structure, representing an inner–outer spatial relationship, is a fundamental double-chamber structure that has received significant attention in the literature for developing novel functional nanomaterials and for providing the desired drug sustained-release profiles [39,40,41].

Just as with monolithic nanoproducts, core–shell nanostructures can be created through either a bottom-up route, such as molecular self-assembly, or a top-down manner [42,43,44]. Electrohydrodynamic atomization (EHDA) is a typical top-down method that capitalizes on the favorite interactions between the working fluids and electrostatic energy to replicate the configuration of the spinneret or spraying head [45,46,47,48,49,50]. EHDA fabrications are always carried out in a single-step and straightforward manner, thus holding great promise for the industrial-scale productions of nanoproducts [51,52,53]. Therefore, its branched techniques, such as coaxial electrospinning and electrospraying, have been widely used to create various core–shell nanofibers or nanoparticles, marking significant progress in the field [54,55,56].

The first application of electrospun nanofibers is about drug sustained release [36]. Similarly, the first application of electrospun core–shell nanofibers was also aimed at providing drug sustained-release profiles [57]. Electrospun core–shell nanofibers can be employed as a powerful support for developing numerous functional nanomaterials for a wide variety of applications, including drug sustained-release materials [58,59,60,61]. To date, many strategies have been reported for exploiting electrospun and electrosprayed core–shell structures to control the sustained-release behavior of drug molecules [62,63]. One strategy is to set up a shell barrier to retard the release of drug molecules, and thus to achieve a sustained-release profile [64]. The barriers include the insoluble cellulose acetate, zein, ethylcellulose, and also lipid [65,66]. Another strategy involves tailoring the core components, such as drug depots as the cores; loading drug-contained hydrophobic nanoparticles in the core; and co-loading drugs with inorganic or other insoluble additives [67,68]. Most strategies that utilize core–shell nanostructures focus on either the core or the shell separately. Very limited attention has been paid to simultaneously manipulating both the core and the shell sections in a systematic manner.

Ferulic acid (FA), a poorly water-soluble drug, originally discovered in plant seeds and leaves, is a phenolic acid widely present in the plant body. It binds with polysaccharides and proteins in the cell wall to form the skeleton of the cell wall and rarely exists in a free state [69]. It is one of the effective ingredients in Chinese medicinal herbs such as *Ferula*, *Cimicifuga*, *Angelica sinensis*, and *Ziziphus jujuba* [70]. FA has good pharmacological effects and biological activity, and therefore has high application value in medicine, health products, and cosmetic raw materials [71]. It has been reported that FA possesses various beneficial effects, including antioxidant, antibacterial, and anti-inflammatory activities, as well as anti-thrombotic, anticancer, and anti-radiation properties [72,73]. It also helps prevent lipid oxidation, platelet aggregation, and thrombosis formation, while improving cardiovascular and cerebrovascular health and preventing bone loss. Sustained release is crucial for its efficacy.

Based on the above-mentioned background, here, we hypothesize that a systematic manipulation of the core and the shell sections within the electrospun nanofibers may comprise a new way for developing sophisticated core–shell products with an improved functional performance. The new core–shell structures were designed to have a radical decrease distribution of insoluble ethylcellulose (EC) from the shell to core sections, and also a radical increase distribution of soluble polyvinylpyrrolidone (PVP) from the shell to core sections. To verify the usefulness of these radical distributions of PVP and EC, FA was homogeneously distributed throughout the nanofibers.

## 2. Materials and Methods

### 2.1. Materials

Ethylcellulose (EC, 6 mPa•s to 9 mPa•s), polyvinylpyrrolidone (PVP) K60 (Mw = 360,000), methylene blue, anhydrous ethanol, acetone, and *N*,*N*-dimethylacetamide (DMAc) were bought from Sigma-Aldrich (Shanghai, China). Ferulic acid (purity: 98%) was purchased from Shanghai Haosheng Biomed. Co., Ltd. (Shanghai, China). A phosphate buffer solution (PBS, 0.1 M, pH = 7.0) was supplied by Tianjin Zhiyuan Chemical Reagent Co., Ltd. (Tianjin, China). All chemicals and reagents were used without any additional treatment and water was double-distilled just before use.

### 2.2. Electrospinning

A homemade electrospinning apparatus was explored for all the preparations, which was characterized by a homemade concentric spinneret. The other elements of the apparatus included two syringe pumps (one KDS 100 and one KDS200, Cole-Parmer, Holliston, MA, USA), a ZGF60kV/2mA high-voltage generator (Wuhan Hua-Tian High Power Co, Ltd., Wuhan, China), and a simple collector prepared from the aluminum foil and a hard cardboard. The experimental parameters are included in Table 1. 

### 2.3. Morphologies and Inner Structures

A scanning electron microscope (SEM, FEI Quanta 450 FEG, FEI Corporation, Hillsboro, OR, USA) was employed to observe the surface morphologies of all the nanofibers. All the samples were subjected to Au-coating for 1 min before they were placed into the SEM chamber. And the diameter distribution statistics were obtained by using the ImageJ software V1.8.0 (NIH, Bethesda, MD, USA) and Origin 2021 for diameter distribution statistics.

A transmission electron microscope (TEM, Thermo Talos F200X G2, Waltham, MA, USA) was employed to assess the inner structure of the resultant nanofibers. The samples were prepared by placing a copper-supported carbon film under the spinneret but just above the collector to collect samples for about 10 s.

### 2.4. Physical State and Compatibility

The components’ physical states were assessed using a Bruker D8 Advance X-ray diffractometer (XRD, D8 ADVANCE, Bruker, Germany) with a copper target tube and under a voltage of 40 kV, a tube current of 40 mA, a scanning rate of 8°/min, and a minimum step of 0.02°. Attenuated total reflectance Fourier transform infrared spectroscopy (ATR-FTIR) was performed using a SPECTRUM 100 spectrometer (Perkin Elmer, Waltham, MA, USA). The resolution of the instrument was 1 cm^−1^, the scanning range was 450–4000 cm^−1^, and the scanning times were 8.

The differential scanning calorimetry (DSC) assessments of the raw materials and electrospun nanofibers F1, F2, and F3 were conducted using a DSC instrument (MDSC 2910, TA Instruments Co., New Castle, DE, USA). Sealed samples were heated at 10 °C·min^−1^ from the ambient temperature to 220 °C. The nitrogen gas flow rate was 40 mL·min^−1^.

### 2.5. In Vitro Dissolution Tests

In vitro dissolution tests of the blended nanofibers F1 and F2, and core–shell nanofibers F3, were carried out to assess the drug sustained-release profiles. In vitro dissolution tests were performed according to the paddle method described in the Chinese Pharmacopoeia (2020 Ed.). An amount of nanofibers containing 20 mg FA was immersed in the vessels of a dissolution apparatus (RCZ-8A, Radio Factory, Tianjin University, Tianjin, China), which contained 900 mL of PBS at 37 °C and with a rotation speed of 50 rpm. At pre-determined intervals, 5.0 mL aliquots of the release media was collected from the dissolution vessels and 5.0 mL fresh PBS was compensated for keeping a constant volume. The FA absorbances were measured using a UV-vis spectrophotometer (Unico2000, Unico Co., Ltd., Shanghai, China) at a wavelength of 320 nm. The pre-determined standard equations between absorbance (A) and drug concentration (C, μg/mL) were employed to calculate the concentrations of FA, A = 0.5764 × C − 0.0236 (R = 0.9997, range: 1 to 50 μg/mL). The accumulative release percentage, i.e., P (%), can be further calculated based on the following Equation (1) [25]:

(1) $$P(\%)=\frac{C_{n}\times V_{0}+\sum_{i=1}^{n-1}C_{i}\times V}{Q_{0}}\times 100$$

where V_0_ is the volume of the dissolution medium (900 mL), V is the volume of the sample drawn (5.0 mL), Q_0_ is the theoretical amount of the drug in each sample (mg), C_n_ is the concentration of the drug measured in the nth aliquot (mg/L), and C_i_ is the concentration of the drug in the ith aliquot (mg/L).

### 2.6. Statistical Analysis

The data were expressed as the means ± standard deviations (S.D.). The statistical analysis of the data was performed using an analysis of variance (ANOVA). Where necessary, the ANOVA was followed by Dunnett’s test, with a *p*-value of <0.05 considered statistically significant.

## 3. Results and Discussion

### 3.1. Strategies for Developing Novel Core–Shell Nanostructures with Radical Distributions of Soluble and Insoluble Polymers

**Figure 1:**
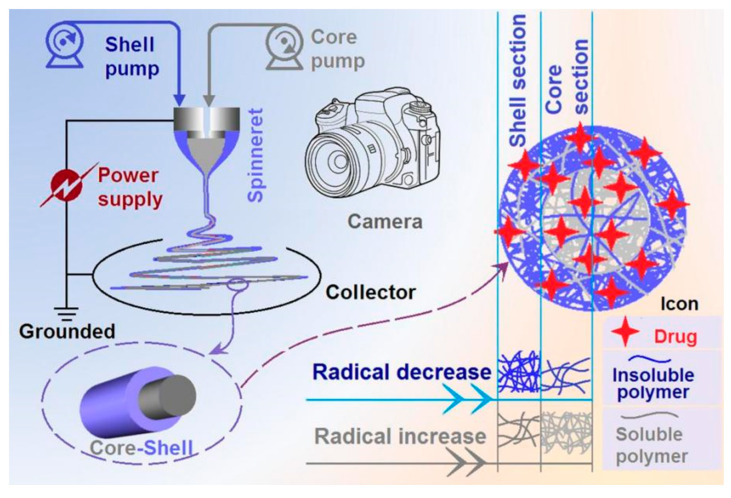
A diagram of the coaxial electrospinning apparatus, the electrospun core–shell nanofibers, and the brand-new strategy of tailoring the radical distributions of soluble and insoluble polymers for achieving a high-quality sustained-release profile.

The popularity of core–shell nanostructures has greatly promoted the development of coaxial electrospinning [42,56,74]. Vice versa, the capability of coaxial electrospinning in treating different kinds of working fluids has greatly enriched the strategies of developing novel functional nanomaterials based on core–shell nanostructures [75,76,77]. Like traditional blending electrospinning and bi-fluid side-by-side electrospinning [78,79,80], a coaxial electrospinning apparatus consists of four fundamental components, as shown in Figure 1: one or two pumps (one for blending electrospinning and two for coaxial electrospinning) to drive the working fluids quantitatively, a power supply, a spinneret, and a collector. Other attachments include a camera for the observation of the working process and an auxiliary drying apparatus.

Coaxial electrospinning has been demonstrated to be able to create core–shell nanofibers, high-quality monolithic nanofibers with a solvent as a shell fluid, and tri-layer core–shell nanofibers [81,82,83,84]. Nonetheless, the mainstream is the double-layer core–shell ones. By manipulating the components in the shell and core sections, core–shell nanofibers can easily be tailored to provide a variety of desired functional properties. However, the core and shell sections can have the same components but with various compositions. This is a new standpoint for developing novel core–shell nanostructures. As shown in Figure 1, both core and shell sections contain the same kind of soluble polymer, the same kind of insoluble polymer, and the same drug molecules. However, their compositions vary. The insoluble polymer has a significantly decreased distribution from the outer shell to the inner core, while the soluble polymer exhibits a simultaneous radical increase in distribution from the shell to the core. The impact of these reverse gradient distributions of soluble and insoluble polymers on the sustained-release behaviors of drug molecules will be revealed through various characterization techniques.

### 3.2. Successful Implementations of the Electrospinning Processes

**Figure 2:**
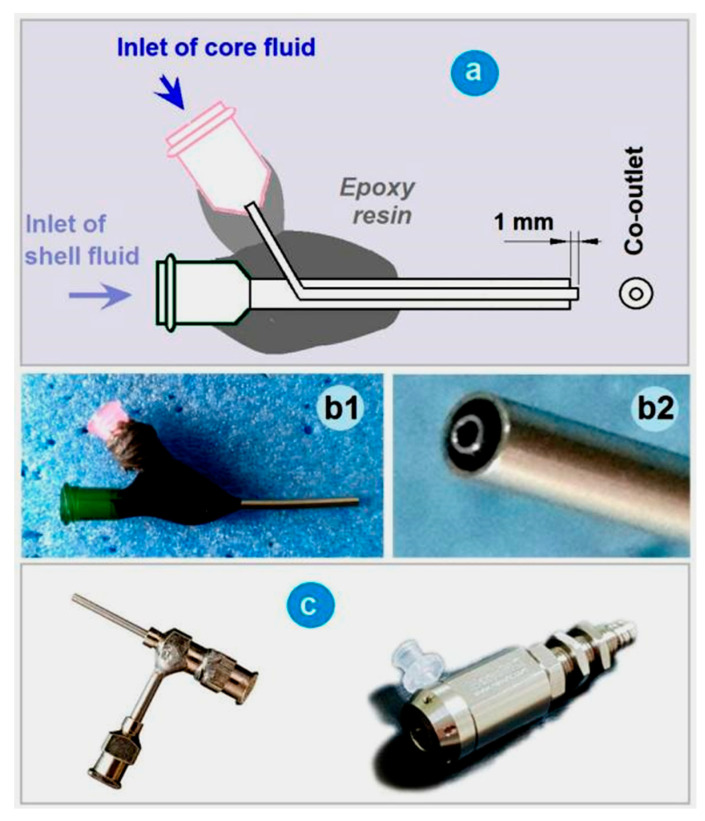
A concentric spinneret for coaxial electrospinning: (**a**) A diagram showing the parameters for fabricating the homemade concentric spinneret, the fixing and connection of the different parts, and the inserting of inner stainless steel capillaries. (**b1**,**b2**) Digital images of the whole concentric spinneret, and the co-outlet of the spinneret’s nozzle, respectively. (**c**) Two concentric spinnerets commercially available.

Among the four fundamental sections of an electrospinning apparatus, the spinneret is the most important one and is a hub of innovation [85,86,87]. The electrospinning process is often categorized and named by the spinneret, such as coaxial electrospinning, which involves a concentric spinneret where the core and shell capillaries share a common axis, or side-by-side electrospinning, which is facilitated by an eccentric parallel spinneret. In this study, a homemade spinneret was employed for conducting all the electrospinning processes. A diagram showing the parameters for fabricating the homemade concentric spinneret, the fixing and connections of different parts, and the inserting of inner stainless steel capillaries is exhibited in Figure 2a. The digital images of the whole concentric spinneret, and the co-outlet of the spinneret’s nozzle, are shown in Figure 2(b1,b2), respectively. For comparison, the digital images of two concentric spinnerets commercially available are shown in Figure 2c.

The homemade concentric spinneret has three distinctive properties for implementing electrospinning: (1) a combination of a polymer (epoxy resin and rubber) and stainless steel capillaries, and most of the spinneret surface is covered by the insulated polymers and thus is conducive for energy saving [88], and the commercial concentric spinnerets have a large metal surface to lose the electrostatic energy to the environment; (2) a very light weight, only 2.1 g, compatible for setting up the electrospinning apparatus—a heavy spinneret may even result in falling off from the syringe and the related safe operation issue; and (3) the core metal capillary is slightly projected out at 1.0 mm from the shell capillary, and this arrangement is favorable for the fine encapsulation of core fluid by the shell fluid.

**Figure 3:**
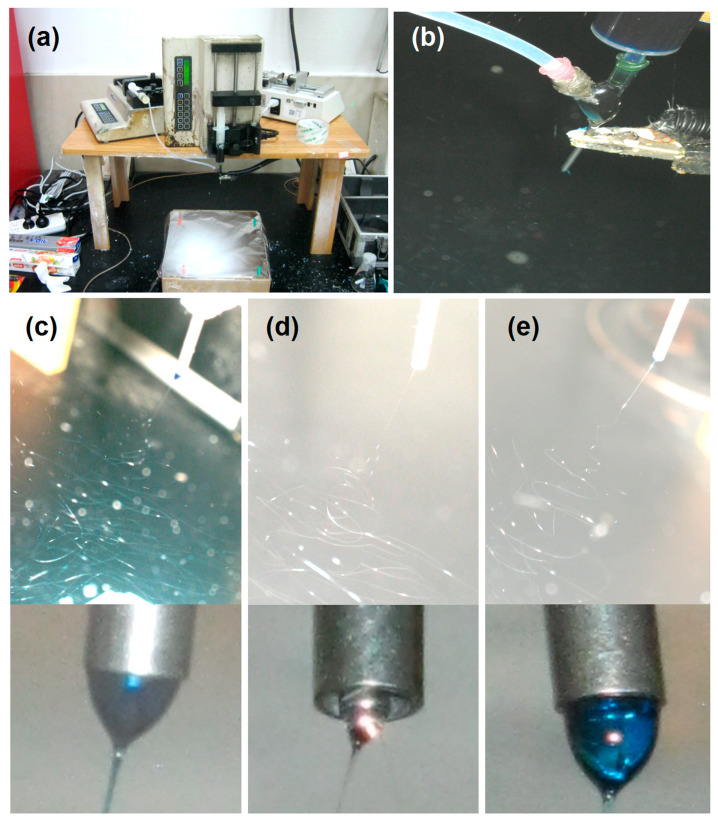
Implementations of the three electrospinning processes for creating the three kinds of nanofibers: (**a**) An aerial view of the whole coaxial electrospinning apparatus with two pumps and a power supply. (**b**) The convergent point of two working fluids and electrostatic energy transferring. (**c**) A typical working process for preparing the homogeneous nanofibers F1 from the shell fluid with methylene blue as a color marker; the bottom inset is an image of the blue Taylor cone. (**d**) A typical working process for preparing the homogeneous nanofibers F2 from the core fluid; the bottom inset is an image of the transparent Taylor cone. (**e**) A typical working process for preparing the core–shell nanofibers F3; the bottom inset is a typical image of the compound core–shell Taylor cone.

An aerial view of the whole coaxial electrospinning apparatus with two pumps and a power supply is shown in Figure 3a, which was running for preparing the core–shell nanofibers F2 (the shell fluid was switched off). During the working process, the spinneret is the key place, as indicated by Figure 3b. It is the convergent point of two working fluids that were led to the electrical field and the transferring of electrostatic energy by an alligator clip. Meanwhile, this place was also the starting point for beginning a working procedure. With methylene blue as a color marker in the shell section (10^−6^ g/L), the working processes for fabricating the three kinds of nanofibers can be clearly recorded by a digital camera (Canon G7x, Tokyo, Japan). As indicated in Figure 3c–e, respectively, all the single-fluid electrospinning from only the shell capillary or the core capillary, and the double-fluid coaxial electrospinning, had the typical three successive steps, i.e., the Taylor cone, the straight fluid jet, and the instable regions full of bending and whipping loops with a gradual increase in their diameters. Their Taylor cones are given in their corresponding bottom insets. A whole blue Taylor cone of the shell solution is shown in the bottom inset of Figure 3c, and a transparent small Taylor cone of the core fluid is shown in the bottom inset of Figure 3d. For the coaxial process, the Taylor cone was a compound core–shell one (the bottom inset of Figure 3e), with the outer shell blue fluid encompassing the inner core transparent fluid.

### 3.3. The Morphologies and Inner Structures of the Electrospun Nanofibers

**Figure 4:**
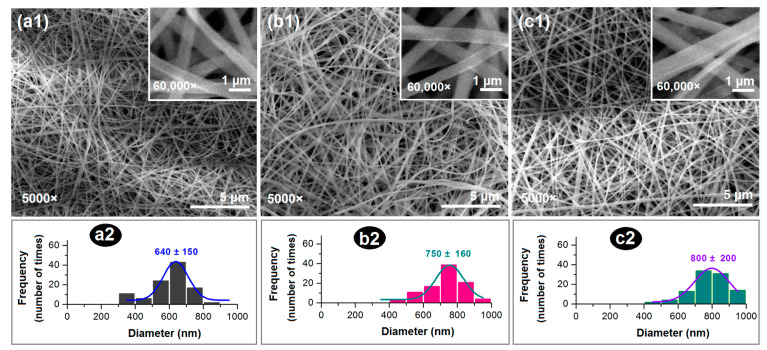
The morphologies of the prepared nanofibers and their diameter distributions: (**a1**,**a2**) Monolithic nanofibers F1. (**b1**,**b2**) Monolithic nanofibers F2. (**c1**,**c2**) Core–shell nanofibers F3; the **upper-right** insets are the enlarged SEM images of the corresponding nanofibers.

As shown in Figure 4(a1–c1) and their corresponding enlarged insets, all the nanofibers had a fine linear morphology without any discernible spindles or beads, suggesting that both working fluids have good electrospinnability and are compatible with each other. The diameter distributions of nanofibers F1, F2, and F3 are shown in Figure 4(a2–c2), respectively. The diameters of core–shell nanofibers F3 were slightly larger than those of the homogeneous nanofibers F2 and F1. This gives a hint that the interfacial tensions between the core and shell fluids have played their important roles in the formations of core–shell nanostructures [62], which warrants further investigation in future studies.

**Figure 5:**
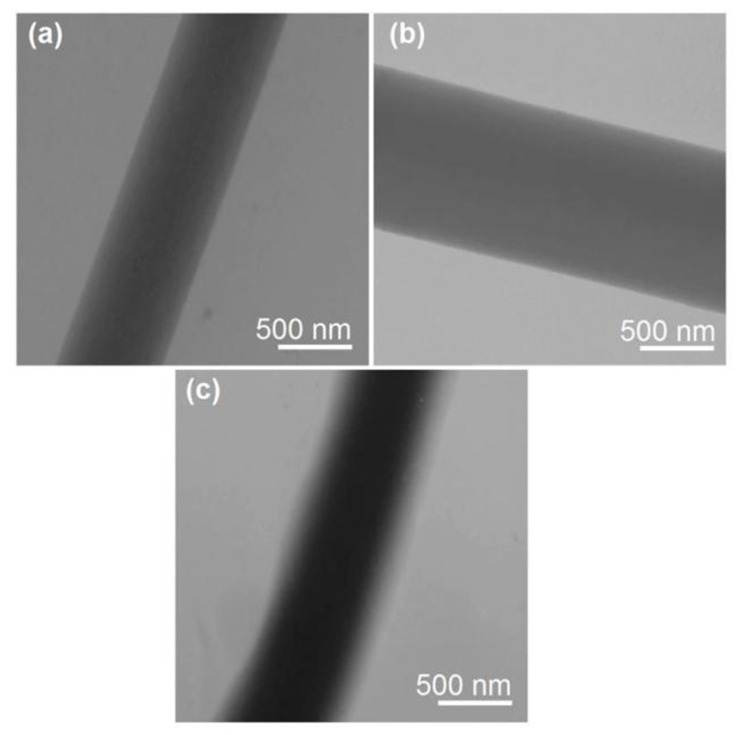
TEM images of the prepared nanofibers: (**a**) monolithic nanofibers F1; (**b**) monolithic nanofibers F2; and (**c**) core–shell nanofibers F3.

The TEM images of the monolithic nanofibers F1 and F2, as well as the core–shell nanofibers F3, are shown in Figure 5. As expected, both nanofibers F1 and F2 had a uniform gray level, indicating homogeneous and monolithic nanostructures. In sharp contrast, the core–shell nanofibers F3 exhibited distinct gray levels of the core and shell sections (Figure 5c). This can be attributed to the fact that the core sections definitively had a larger thickness and possibly a higher density, even though their boundaries were not very distinct. Estimations from Figure 5c indicated that the shell sections had an average thickness of 140 ± 34 nm, while the core sections had an average value of 560 ± 21 nm. Thus, the density ratio of the core section to shell section can be calculated, which should be inversely proportional to their surface area ratio, i.e., {[(280 + 140)^2^π-(280^2^π)]/(280^2^π)} × 100% = 125%, indicating that the core composites had a relatively higher packing density.

### 3.4. The Physical State of FA and Its Compatibility with EC and PVP

**Figure 6:**
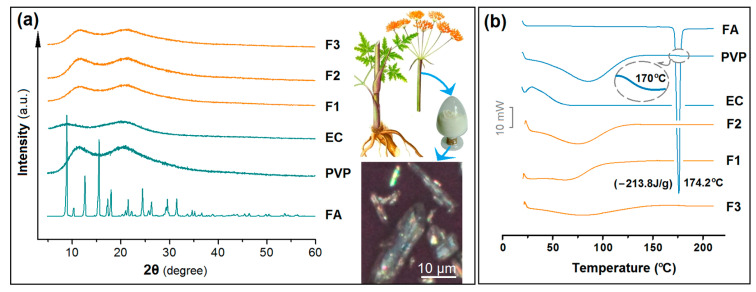
Physical state characterizations: (**a**) XRD patterns of the starting materials (PVP, EC, and FA) and their nanofibers F1 and F2 and their core–shell nanofibers F3; the plants for extracting FA, its right powders, and their polarized microscopic image. (**b**) DSC curves of FA, PVP, EC, and their composite nanofibers F1 to F3.

For the poorly water-soluble drugs, the amorphous state is better than their crystal state for effective dissolution and controlled release. Within the electrospun nanofibers, the high homogeneous state of drug molecules in the working fluid can be propagated into the solid nanofibers due to the extremely fast drying process of electrospinning. XRD is frequently explored to reveal the physical state of components within nanoproducts [89,90]. The XRD patterns of the initial raw materials of EC, PVP, and FA and their nanofibers F1, F2, and F3 are exhibited in Figure 6a. As expected, the drug FA has a series of sharp Bragg peaks at its patterns, indicating its crystalline state in the raw powder form, which is primarily extracted from the plant of *Ferula asafoetida* and has a light yellow color. Its crystalline state in the raw form can also be confirmed by polarized light microscopy. As shown in the right section of Figure 6a, the particles exhibit red, blue, green, and yellow colors on their surface, resulting from the different crystal planes of the crystalline FA powders. Both PVP and EC exhibit amorphous characteristics, as evidenced by their broad halos in the XRD patterns. When they were converted into homogeneous nanofibers F1 and F2, or core–shell nanofibers F3 with radical distributions of polymers, they all retained an amorphous state. This should be an extension of the highly dispersion state they presented in their working fluids, instantly solidified by the interactions between the working fluids and the electrostatic energy.

A further DSC analysis was conducted to determine the physical state of the components in the three types of nanofibers. The DSC thermograms are shown in Figure 6b. The DSC thermogram of pure FA exhibited a single endothermic response at 174.2 °C. Both PVP and EC, being amorphous polymers, do not exhibit sharp endothermic peaks. Interestingly, a small transition from a glassy to a rubbery state in PVP is observed between 160 and 180 °C (as shown in the inset of Figure 6b). This transition state is not discerned in the DSC thermograms of the three types of nanofibers. These phenomena jointly suggest that FA was no longer present as a crystalline material but had been converted into an amorphous state within all the nanofibers.

For the stable storage and shipping of dosage forms containing poorly water-soluble drugs, fine compatibility between the drug molecules and the polymeric matrices is crucial [10,15,91]. ATR-FTIR spectra can be employed to assess the compatibility between FA and both EC and PVP.

**Figure 7:**
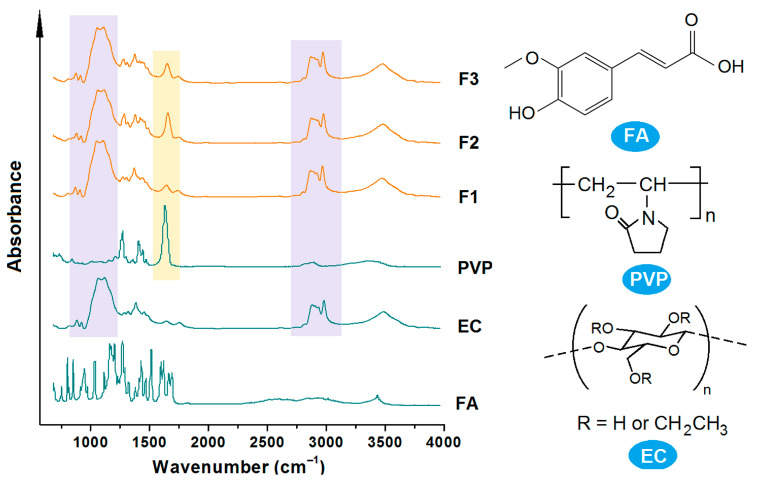
ATR-FTIR spectra of the starting materials (PVP, EC, and FA) and their nanofibers F1 and F2 and their core–shell nanofibers F3, and their molecular formula.

The spectra of the raw materials—EC, PVP, and FA—as well as their three kinds of electrospun nanofibers F1 to F3 are included in Figure 7. As indicated by their molecular formula, FA molecules have two OH groups and one C=O group, PVP has many C=O groups within a molecule, and EC has many OH groups in one molecule. The presence of these groups suggests that hydrogen bonds can be extensively formed between FA and PVP, FA and EC, and PVP and EC, indicating that these components are highly compatible. The spectra of the three nanofibers clearly demonstrate these interactions, with apparent overlapping peaks from PVP and EC. As indicated by its molecular formula, the chemical structure of FA consists of one aromatic ring, which is facile for forming a large π electron cloud with the long carbon chains within EC and PVP, by which the characteristic peaks in the spectra of FA disappeared from those nanofibers’ spectra.

### 3.5. The In Vitro Dissolution Profiles of FA from the Three Kinds of Nanofibers

**Figure 8:**
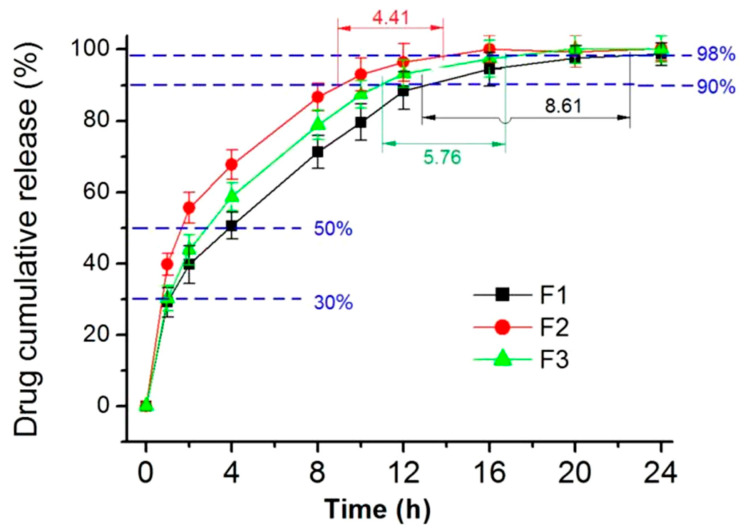
The in vitro dissolution profiles of the homogeneous nanofibers F1 and F2, and the core–shell nanofibers F3.

As expected, all the three types of electrospun nanofibers, F1, F2, and F3, provided a typical sustained-release profile of FA, as indicated in Figure 8. To further disclose the differences of their drug sustained-release profiles, the interpolation method was used to determine the times that were required to release specific percentages of the loaded FA. The percentages of 30, 50, 90, 95, and 98% were used to evaluate the drug sustained performances, and particularly for assessing the initial burst release, the extended-release time period, and the severity of tailing-off release. The results are included in Table 2.

Compared with the homogeneous nanofibers F1 and F2, the core–shell nanofibers F3 were able to furnish the best FA sustained-release profiles. This can be judged from the following aspects: (i) a smaller initial burst release for F3 compared to F1 and F2, with values of approximately 1.06 and 1.12 being greater than 0.75; (ii) taking the longest middle time period to reach the 50% release, with times of 3.92, 2.86, and 1.74, which indicates a moderate effect in extending the continuous release time period of FA; (iii) having a moderate time range from reaching 90% to 95% release of the loaded FA, i.e., 3.84 > 2.82 > 1.92, and similarly, from 90% to 98% release, with times of 8.62, 5.76, and 4.41, giving a hint of weakening the tailing-off release by the inner core section with a higher content of PVP. Combining these data indicates that the core–shell nanofibers F3, with radical distributions of soluble polymers and insoluble polymers, on one hand, effectively prevented the initial burst release, similar to nanofibers F1, which have a higher content of insoluble EC. On the other hand, they inhibited the negative tailing-off release, much like the homogeneous nanofibers F2, which have a higher content of soluble PVP. In other words, the core–shell nanofibers F3 were able to combine the advantages of both F1 and F2.

### 3.6. The Drug Sustained-Release Mechanisms from the Medicated Nanofibers

Sustained release is the most popular drug controlled-release profile for treating a series of diseases [92,93,94,95]. The Peppas equation, Equation (2), is frequently employed to reveal the drug release mechanisms from their polymeric matrices, where Q represents the cumulative drug release percentage, t represents the needed time, and n is an indicator of the drug release mechanism [96].

Q = k× t^n^ (2)

It is well known that an “n” value smaller than 0.45 suggests a Fickian diffusion mechanism, a value larger than 0.89 indicates an erosion mechanism, and a value between 0.45 and 0.89 signifies a combination of diffusion and erosion mechanisms.

**Figure 9:**
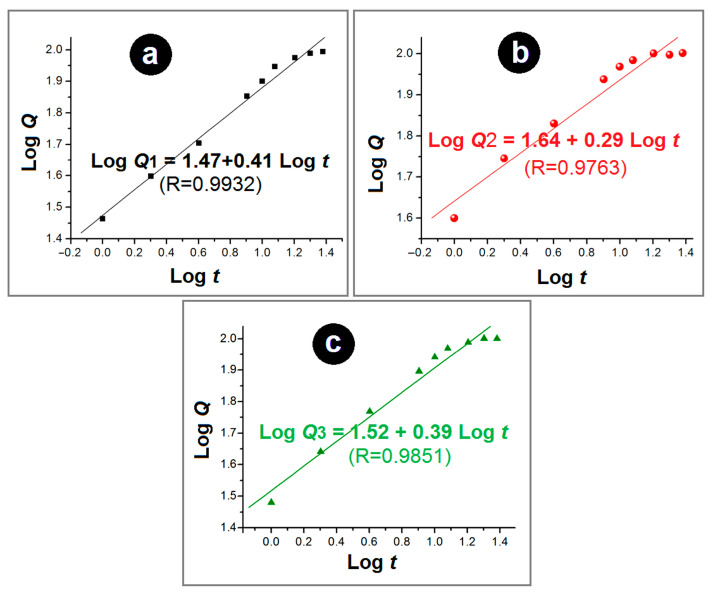
The drug release mechanisms of nanofibers (**a**) F1, (**b**) F2, and (**c**) F3, regressed according to the Peppas equation.

Using the Peppas equation and the in vitro dissolution data, the regression equations for nanofibers F1, F2, and F3 could be determined to be LogQ1 = 1.47 + 0.41Logt (R = 0.9932), LogQ2 = 1.64 + 0.29Logt (R = 0.9763), and LogQ3 = 1.52 + 0.39Logt (R = 0.9851), as shown in Figure 9a–c, respectively. The “n” values of the monolithic nanofibers F1 and F2 and the core–shell nanofibers F3 were 0.41, 0.29, and 0.39, respectively. Since all these values are below the critical value of 0.45, this suggests that FA molecules were released from all the nanofibers via a typical Fickian diffusion mechanism, irrespective of monolithic or core–shell nanostructures.

**Figure 10:**
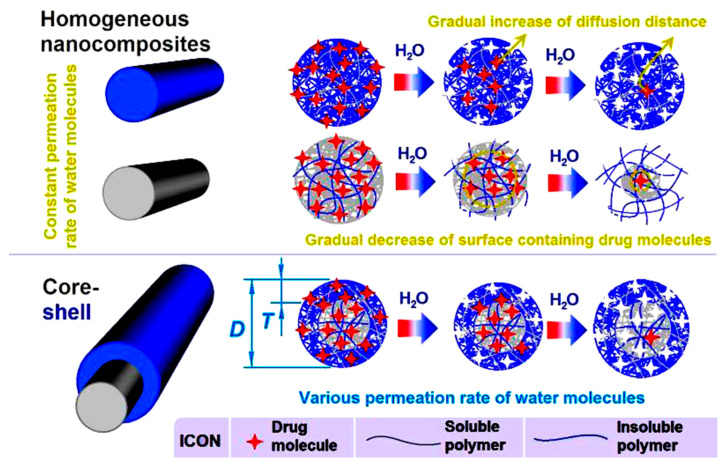
A diagram about drug molecule release behaviors from the homogeneous nanofibers F1 and F2, and the core–shell nanofibers F3.

To further disclose the drug molecule release behaviors from the three kinds of nanofibers, a diagram is presented in Figure 10. The homogeneous nanofibers F1 and F2 are monolithic nanocomposites. They contain the same components (FA, EC, and PVP) with uniform compositions throughout their inner cross-sections and surfaces, resulting in similar physical properties. The water permeation rate from their surfaces of nanofibers F1 and F2 to their inner sections, and ultimately to the central axes, would be similar, progressing in line with the in vitro dissolution tests. Thus, an initial burst release is inevitable due to the largest drug-contained surface areas, the shortest distance of water molecule permeation and drug molecule diffusion, and the unique properties of electrospun nanofibrous mats (such as small diameter, large surface, and big porosity). Meanwhile, the increased presence of water-soluble polymer PVP in nanofibers F2 enhances the hydrophilicity of the nanofibers and the permeation rate of water molecules, which in turn exacerbates the initial burst release compared to nanofibers F1.

Later, FA molecules gradually diffuse from the swollen nanofibers into the bulk solution as sufficient water molecules permeate to facilitate their transfer. By the end of in vitro dissolution, only drug molecules at the very center of nanofibers remain within the solid matrix. At that point, the drug-containing surface area is smaller than in previous stages, and the diffusion distances are longer than in previous stages. Thus, it is inevitable that a long time period of tailing-off negative release appears. This tailing-off release cannot offer a drug concentration over the minimum blood concentrations for effective therapy, and thus means a waste of the drugs and should be avoided. Meanwhile, an increase in the water insoluble polymer in nanofibers F1 means a narrow water molecule permeation and drug molecule diffusion route, and in turn an even longer time period of tailing-off release, i.e., an exacerbation of the negative effect of the final drug release.

PVP is highly soluble in water and is able to increase the solubility of many insoluble drugs [97]. When the radical distributions of soluble and insoluble polymers are built within the electrospun core–shell nanofibers, the shell sections would have a smaller water permeation rate due to a higher content of EC but a small content of PVP. Conversely, the core sections would have a larger water permeation rate due to a higher content of PVP but a small content of EC. Thus, at the beginning of in vitro dissolution tests, when there is the largest drug-contained surface area and the shortest diffusion distance, a lower water permeation rate due to the higher content of EC and lower content of PVP would be useful for effectively limiting the initial burst release. As the dissolution process reaches the core section, the increased permeation rate of water molecules, resulting from a higher content of PVP and a lower content of EC, would create an inner drug “reservoir” with a saturated concentration of drug molecules. The benefits include at least the following two aspects: First, it provides a higher drug concentration difference between the inner of solid fibers and the surrounding bulk solution, promoting constant and continuous drug molecule diffusion, i.e., stable sustained-release behavior. Second, it shortens the time and distance for water molecules to reach the innermost drug molecules, thereby inhibiting the tailing-off negative release. In summary, core–shell nanofibers can combine the advantages of composite nanofibers with a higher content of water-insoluble polymers to retard the initial burst release and extend the drug release period, along with the advantages of composite nanofibers with a higher content of water-soluble polymers to inhibit the tailing-off release at the end of dissolution. The key fundamental element is the water molecule permeation rate, which can exert a significant influence on the dissolution and diffusion of drug molecules uniformly distributed throughout the solid nanofibers.

The new strategy, a variable ratio of soluble-to-insoluble polymers within the electrospun core–shell structure, not only paves a new way for developing a wide variety of polymeric composites with heterogeneous distributions to achieve desired functional performances, but also serves as a reference for developing new kinds of functional materials based on electrosprayed core–shell/Janus microparticles [98], electrospun Janus nanofibers [99,100], tri-chamber core–shell and tri-section Janus structures [101,102], and the complex combinations of Janus and core–shell structures [62]. Certainly, various parameters of the core–shell nanofibers can be further manipulated to adjust the drug sustained-release profiles. Firstly, the thickness of the shell section can be controlled through changing the shell-to-core fluid flow rate ratio, which in turn affects the drug sustained-release profiles and the related process–structure–performance relationship. Secondly, adjusting the ratio of insoluble-to-soluble polymers during the preparation of electrospinnable working fluids can further refine the sustained-release profile and establish a specific process–structure–performance relationship. Thirdly, the drug distribution within the core–shell nanofibers can be manipulated, potentially creating a nanodrug depot for sustained release. Fourthly, the overall diameter of the core–shell nanofibers can be controlled by adjusting operational parameters and the properties of the working fluids, thereby regulating drug sustained release. Certainly, the judicious selection of polymeric matrices [103,104,105,106] and the use of modern tools, such as molecular simulation [107], can further enrich these strategies significantly.

## 4. Conclusions

In this study, a coaxial electrospinning was conducted to prepare a brand-new type of core–shell nanofibers using a homemade concentric spinneret and the coaxial electrospinning apparatus. Based on the electrospinnability of the shell fluid with a higher concentration of EC over PVP and the core fluid with a lower concentration of EC over PVP, the coaxial processes could be conducted robustly and continuously. All three types of nanofibers—homogeneous F1 from the shell fluids, homogeneous F2 from the core fluid, and heterogeneous core–shell nanofibers F3—exhibited a fine linear morphology without discernible beads or spindles, as demonstrated by their SEM images. The core–shell nanofibers F3 had radical distributions of both EC (increasing from the shell to the core) and PVP (increasing from the core to the shell). TEM observations verified the monolithic structures of F1 and F2, as well as the core–shell structures of F3. XRD measurements indicated that FA was present in a favorable amorphous state in all the nanofibers, regardless of them being homogeneous or heterogeneous. The ATR-FTIR analysis revealed good compatibility between FA and its polymeric matrices. In vitro dissolution tests demonstrated that the radical distributions of EC and PVP played their important roles in adjusting the release behavior of FA molecules. The core–shell nanofibers F3 had the advantages of homogeneous composite nanofibers F1, which had a higher content of EC prepared from the shell solutions, to inhibit the initial burst release and provide a longer time period of sustained release. Additionally, F3 had the advantages of nanofibers F2, which had a higher content of PVP prepared from the core solutions, to inhibit the tailing-off release. Although the Peppas regression equations of the three types of nanofibers indicated that the release of FA from all the nanofibers was inevitably controlled by the typical Fickian mechanisms, the key element was disclosed to be the water permeation rates, which were controlled by the ratios of soluble and insoluble polymers.

## Figures and Tables

**Table 1:** Parameters of the experimental operations and the nanofibrous products.

Sample No.	Electrospinning Process	Operational Parameters ^a^				Drug Content (%)
		V (kV)	F (mL/h)		L (cm)	
			Core	Shell		
F1	Blending	11	-- ^b^	2.0	20	20%
F2	Blending	11	2.0	-- ^b^	20	20%
F3	Coaxial	11	1.0	1.0	20	20%

^a^ Fluid 1 (or the shell fluid) was composed of 15% (*w*/*v*) EC, 3% (*w*/*v*) PVP, and 4.5% (*w*/*v*) FA in a mixture of DMAc and ethanol with a volume ratio of 1:9, while the 2nd fluid (or core fluid) consisted of 7.5% (*w*/*v*) EC, 6% (*w*/*v*) PVP, and 3.375% (*w*/*v*) FA in the same kind of solvent mixture as fluid 1. ^b^ The symbol “--” represents none.

**Table 2:** Evaluation of sustained release in terms of initial burst release, sustained-release effects, and the negative tailing-off release.

Sample	Initial Release	Sustained Release	Tailing-Off Negative Effect				
	t_30%_	t_50%_	t_90%_	t_95%_	t_98%_	t_95%_−t_90%_	t_98%_−t_90%_
F1	1.12	3.92	12.94	16.78	21.56	3.84	8.62
F2	0.75	1.74	9.26	11.18	13.67	1.92	4.41
F3	1.06	2.86	10.82	13.64	16.58	2.82	5.76

## Data Availability

Data are contained within the article.
